# Systematic Review and Meta-Synthesis: Coping Strategies of Children, Adolescents, and Young Adults of Parents with a Mental Illness

**DOI:** 10.1007/s10567-025-00540-8

**Published:** 2025-07-26

**Authors:** Franziska Sawitzki, Lina Kinzenbach, Hanna Christiansen, Nicola Großheinrich

**Affiliations:** 1 Department of Social Sciences, Catholic University of Applied Sciences of North Rhine-Westphalia (katho), Campus Cologne, Germany; 2 Department of Psychology, Clinical Child and Adolescent Psychology, Philipps-University Marburg, Marburg, Germany

**Keywords:** Systematic review, Meta-synthesis, Children of parents with a mental illness, Adolescents, Emerging adults, Coping strategies

## Abstract

To examine and integrate the current state of knowledge regarding qualitative literature on the coping strategies employed by children, adolescents, and young adults of parents with a mental illness. A systematic literature search was conducted in November 2023 in the following databases: APA PsycInfo, PubMed, Web of Science, CINAHL Ultimate, and Psychology and Behavioral Science Collection. Peer-reviewed studies utilizing a qualitative or mixed-method design were eligible for inclusion. After quality assessment, a total of 14 articles were included. These studies were analyzed following a qualitative research synthesis, and the reported coping instances were extracted and classified into higher-order families of coping, as conceptualized by Skinner and colleagues (Skinner et al., Psychological Bulletin 129:216–269, 2003). Children, adolescents, and young adults of parents with a mental illness predominantly used support seeking and accommodation as coping strategies. This was followed by strategies such as escape, isolation, and self-reliance. A significant finding was the prominence of role reversal as a coping strategy, which did not fit into the existing system of higher-order families of coping. The established categories for classifying coping in normative samples require modification to better reflect the specific experiences of offspring of parents with a mental illness. Consequently, a new model of coping of COPMI (children of parents with a mental illness) is proposed, building on previous models and integrating findings from this review.

**Supplementary Information:**

The online version contains supplementary material available at 10.1007/s10567-025-00540-8.

## Introduction

In Germany, estimates indicate that around 25% of all children grow up with a parent with a mental illness (Christiansen et al., 2020); the global prevalence is similar (Maybery et al., 2009; Pierce et al., 2020; Reupert et al., 2013). Parental mental illness can have serious consequences for the child’s health, level of functioning and development (Mattejat & Remschmidt, 2008). It can also have long-lasting detrimental effects on emerging adults and later on in adulthood (Kageyama et al., 2023; Källquist & Salzmann-Erikson, 2019). The lifetime risk for developing a mental disorder is significantly increased for offspring of parents with a mental illness compared to the general population (van Santvoort et al., 2015; Zwicker et al., 2023). Mechanisms of this transgenerational transmission of psychopathology have been conceptualized in several models. They all show important interacting risk and resilience factors, which relate to genetics, child, parent, family, or context characteristics (for an overview see Reupert et al., 2015).

**Fig. 1:**
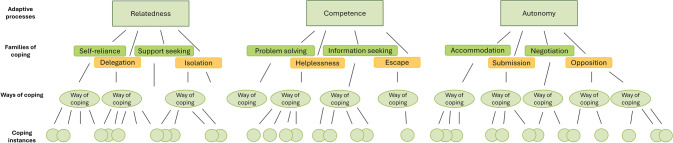
Hierarchical conceptualization of the structure of coping (based on Skinner et al., 2003, p. 218)

In the developmental model of the transgenerational transmission of psychopathology by Hosman and colleagues (2009), coping is one important mediator. Coping can be defined as “an organizational construct that describes how people regulate their own behavior, emotion, and motivational orientation under conditions of psychological distress” (Skinner & Wellborn, 1994, p. 112). As coping can be conceptualized diversely, Skinner and colleagues (2003) conducted a comprehensive review to critically assess these conceptualizations found in the research literature, identifying more than 400 different coping strategies. They organized these strategies hierarchically and evaluated existing categorization systems such as problem- vs. emotion-focused or primary vs. secondary control coping. Continuously, these various conceptualizations were synthesized into a set of 12 higher-order coping categories (Skinner & Wellborn, 1994). This conceptualization is based on a motivational theory of coping, which posits three basic human needs: relatedness, competence, and autonomy. When individuals encounter a stressor that threatens or impinges upon these needs, they experience psychological distress. Such stressor may be perceived as either controllable and appraised as a challenge, or uncontrollable and appraised as a threat (Zimmer-Gembeck et al., 2016). According to Skinner and Wellborn (1994), there are six adaptive coping families for stressors appraised as a challenge (self-reliance, support seeking, problem solving, information seeking, accommodation, and negotiation) and six maladaptive families for when stressors are appraised as a threat (delegation, isolation, helplessness, escape, submission, and opposition). Figure 1 provides a visual depiction of these 12 higher-order coping families based on Skinner et al. (2003, p. 218). In the present review, the hierarchical conceptualization of the structure of coping by Skinner and Wellborn (1994) was used as theoretical framework.

Reviews that integrate the literature on coping in children and adolescents typically focus on coping within the context of normative development and do not specifically address COPMI (e.g., Compas et al., 2017). In one such review by Skinner and Zimmer-Gembeck (2016), problem solving, support seeking, and distraction (an accommodative strategy) were the most commonly reported strategies used by children and adolescents to cope with stress. Distraction is a subtype of the accommodation coping family, which includes processes to adjust to stressful conditions through positive cognitive restructuring or distractive activities (Skinner & Zimmer-Gembeck, 2016, p. 40). Other reviews have specifically focused on coping with certain stressors, such as childhood cancer (Decker, 2006), interparental conflict (Kerig, 2001), academic stressors (Skinner & Saxton, 2019), or parental incarceration (Heinecke Thulstrup & Eklund Karlsson, 2017). While most of these studies have concentrated on coping mechanisms in children and adolescents, only a limited number examined the coping strategies of emerging adults (Jenzer et al., 2019; Kalpidou et al., 2021; Peisch & Burt, 2022). However, as emerging adults often face a wide array of stressors while maintaining close relationships with their parents (Arnett, 2000), we chose not to exclude studies that investigated coping in young adults. Recent research on coping in children, adolescents, and emerging adults highlighted the significant influence of the family context on offspring’s coping (Brady et al., 2023; Francisco et al., 2016; Gervais & Jose, 2020, 2024). In case of a parental mental illness, the parental influence meanwhile can have deleterious effects on children’s coping and subsequently on their mental health.

Concerning coping strategies used by children, adolescents, and emerging adults of parents experiencing mental illness, research findings are inconsistent. Some studies found no significant differences in coping strategies between these individuals and a control group (Buckley & Woodruff-Borden, 2006; Goodday et al., 2019; Jaser et al., 2011; Klimes-Dougan & Bolger, 1998). In contrast, other research identified a differential use of various coping strategies when comparing this target group to offspring whose parents do not have a mental illness (Goodday et al., 2019; Lenz et al., 2011; Mitchell & Abraham, 2018; Vreeland et al., 2019). Additionally, inconsistencies exist in the associations between coping and mental health indicators. For example, some studies found no significant relationship between primary control coping and psychological adjustment (Langrock et al., 2002; Loon et al., 2015; Wong & Power, 2023), a positive association (Anderson et al., 2023; Bettis et al., 2016; Dunbar et al., 2013; Evans et al., 2015; Gruhn et al., 2019; Jaser et al., 2011; Loon et al., 2015), and also a negative one (Jaser et al., 2007). Findings concerning secondary control coping are somewhat more consistent. Except for Langrock and colleagues (2002), who found no significant association between secondary control coping and aggressive behavior problems, most researchers reported a positive link between secondary control coping and psychological adjustment in children, adolescents, and emerging adults of parents with a mental illness (Anderson et al., 2023; Bettis et al., 2016; Compas et al., 2010; Dunbar et al., 2013; Evans et al., 2015; Fear et al., 2009; Gruhn et al., 2019; Jaser et al., 2007, 2011). Similarly, research mainly shows a negative association between disengagement coping and psychological adjustment (Anderson et al., 2023; Bettis et al., 2016; Dunbar et al., 2013; Evans et al., 2015; Goodday et al., 2019; Gruhn et al., 2019; Jaser et al., 2011; Loon et al., 2015; Wong & Power, 2023).

Altogether, qualitative studies have shed light on the coping strategies used by these children, adolescents, and young adults (Petrowski & Stein, 2016; Valdez et al., 2019). Nevertheless, due to methodological limitations, qualitative studies are restricted in their application and generalizability. Therefore, qualitative reviews can help to strengthen their informative value by summarizing individual study results and elucidating potential discrepancies (Walsh & Downe, 2005). However, a systematic review of qualitative literature on this topic has yet to be conducted. The current systematic review aims to address this gap by investigating these research questions:

1. What types of coping instances are reported by children, adolescents, and emerging adults who have a parent with a mental illness?
2. How can these coping instances be categorized into higher-order families of coping?
3. Which reported coping instances relate to challenge and which to threat?

To answer these research questions, evidence from qualitative and mixed-method studies regarding coping strategies of children, adolescents, and young adults of parents with a mental illness were integrated and synthesized.

## Methods

### Protocol and Registration

The present systematic review was registered in and approved by Prospero with the registration number CRD42023463757 (https://www.crd.york.ac.uk/PROSPERO/). The software CADIMA (Kohl et al., 2018) was used as data synthesis tool. This review adheres to the principles outlined in the PRISMA statement for reporting systematic reviews (Page et al., 2021).

### Search Strategy

A systematic literature search was conducted in November 2023 across the following databases: APA PsycInfo, PubMed, Web of Science (all databases), CINAHL Ultimate, and Psychology and Behavioral Science Collection. The most recent search was performed on June 27th, 2025. The search string was elaborated collaboratively by the review team and an expert librarian. It comprised a combination of terms related to parental mental illness, coping strategies of children, adolescents, or emerging adult children and qualitative research, and was tailored to each specific database. The complete search strategy (adapted for Web of Science) is provided in the Supplementary Material, Table S 1.

### Eligibility and Exclusion Criteria

Peer-reviewed qualitative as well as mixed-method studies focusing on the coping strategies of children, adolescents, or young adult children of parents with a mental illness were included in the review. In the case of mixed-method studies, only results from the qualitative part were considered in the analyses. Young adults were defined according to the concept of “emerging adults” as proposed by Arnett (2000); therefore, studies with participants aged up to 30 years were included. If several subsamples with different target groups were investigated within one study, only articles clearly indicating which subsample the respective results belonged to were included. Articles were included only if the total (sub) sample consisted solely of (young adult) children of parents with a mental illness. Papers with mixed samples or where it was unclear whether participants belonged to the group of interest were entirely excluded. Studies were eligible if the diagnoses of the mentally ill parent were precisely stated or listed as examples; studies reporting solely a physical parental illness were excluded, as well as articles focusing only on parental addiction disorder. As coping strategies of offspring of parents with a mental illness were the focus of the present review, articles concerning coping strategies of parents, or any other target group were not reviewed. Moreover, original research papers were included; reviews, meta-analyses, posters, congress or seminar papers, abstracts, theses, and protocols were excluded. For practical reasons, only articles in German or English language were considered. No time or geographical limitations were set in the selection of studies.

### Study Selection

Eligible papers were retrieved through a sequential process, conducted by two reviewers (FS and LK): After screening the title and abstract, full texts of relevant articles were read, and inclusion criteria were verified. Disagreements were resolved by consensus. Reference lists of included studies were checked by the first reviewer (FS) for further additional literature.

### Quality Appraisal

To assess the methodological quality of the included papers, the Mixed Methods Appraisal Tool (MMAT, Hong et al., 2018) was used. This method was chosen due to its applicability to both qualitative and mixed-method studies. First, two general criteria were applied to all included studies, independent of the study type. These criteria involve two screening questions to determine whether clear research questions are present and whether the collected data allow for addressing these questions. Next, for each included study, the appropriate study design category in the MMAT was selected. Qualitative studies were assessed using the five criteria in the qualitative category, while mixed-method studies were evaluated using the five criteria in the mixed-method category. Following the approach of Dobener and colleagues (2022), an overall quality score was calculated for each study, assigning one to five stars based on the number of specific criteria met. One star indicates a study of relatively low quality (20% of criteria met), while five stars indicate a high-quality study (100% of criteria met). No studies demonstrating minor quality were excluded. On the contrary, a more detailed analysis and presentation of the study quality was undertaken to account for possible biases. Two review authors (FS and LK) both assessed independently the quality of the included studies.

### Data Extraction

The following data were independently extracted by the first and second authors (FS and LK): title, publication year, author(s), country, journal, study design, aim, method, parental diagnosis, characteristics of the sample, and coping instances. Coping instances or coping strategies had to be mentioned in the results or discussion section of the respective study. In case of discrepancies between the two reviewers, consensus was reached through discussion. A summary of all extracted data can be found in Supplementary Material, Table S 2.

### Analysis

Based on the data extraction, information regarding the coping instances was synthesized using the conceptualization of higher-order coping families by Skinner et al. (2003), in line with the data synthesis method employed by Mak et al (2021). These higher-order coping families integrate coping strategies and instances at a higher level of abstraction. This facilitates the synthesis of data from different studies, spanning various age groups or employing diverse investigation methods, and thus overcomes issues encountered in previous syntheses (Zimmer-Gembeck & Skinner, 2010, p. 2). For all integrated studies, two authors (FS and LK) independently coded the coping instances, allowing for the assessment of interrater reliability. Discrepancies were resolved through discussions in the doctoral colloquium. Coping instances were assigned to only one coping family; however, in some exceptional cases where a clear assignment was not possible, these instances were classified into two families of coping.

## Results

### Search Outcomes

**Fig. 2:**
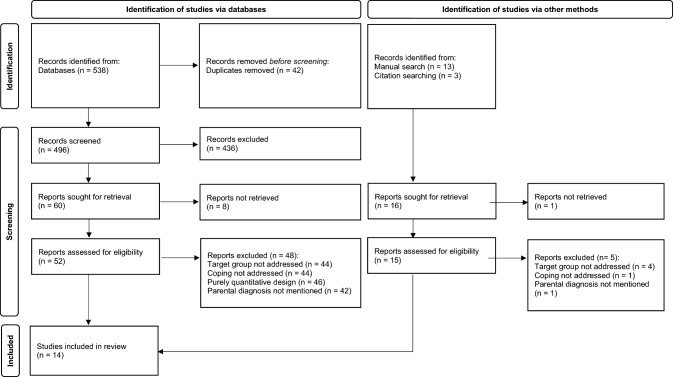
PRISMA flow chart showing study selection

Figure 2 provides an overview of the selection process through the PRISMA flow chart. The initial search yielded 538 articles; after removing 42 duplicates, 496 records were screened. During the first screening phase, 436 records were excluded because of various reasons, such as inappropriate design or type of literature; lack of assessment of coping in children, adolescents or young adult children, or not focusing on the targeted population. Of the 60 articles sought for retrieval, 52 were successfully retrieved. After reading the full texts and excluding 48 additional articles for various reasons (as noted in Fig. 2), four reports remained. Reference list and manual search yielded ten additional articles; in total, 14 articles were included in the present review. A list of excluded studies is available upon request from the corresponding author.

### Study and Population Characteristics

The oldest study included dates back to 1997, while the most recent one was published in 2022. Most of the studies originated from North America or Europe (both 42.9% of all included studies), with only one conducted in Australia and one in Turkey (Asia). Nearly all studies used a qualitative design, merely two mixed-method studies were included. Consequently, in terms of sample size, the majority (78.6%) reported having included up to 20 participants, while only three studies (21.4%) reported more than 20 participants. Regarding sample sex distribution, 64.3% (k = 9) of all studies included only or more female participants than male participants, 14.3% (k = 2) showed an equal split, 7.1% (k = 1) had more male participants, and 14.3% (k = 2) did not mention the sex distribution of their sample. Only one study (7.1%) included children (aged 0–13 years) as participants, three studies (21.4%) included adolescents (aged 14–17 years), and four studies (28.6%) included emerging adults (aged 18–30 years). Additionally, four studies (28.6%) examined both children and adolescents, one (7.1%) focused on adolescents and emerging adults, and one study (7.1%) included participants from all age groups. In terms of parental mental illness, 35.7% of all studies (k = 5) investigated children of parents with an affective disorder, 21.4% (k = 3) focused on children of parents with schizophrenia and most of the studies (42.9%, k = 6) examined several parental disorders. A summary of the study characteristics can be found in Table 1.

**Table 1:** Study characteristics

Characteristics	*k* (% of 14 articles)
Publication date	
1997–2001	2 (14.3)
2002–2006	3 (21.4)
2007–2011	2 (14.3)
2012–2016	4 (28.6)
2017–2022	3 (21.4)
Country of origin	
North America	6 (42.9)
Europe	6 (42.9)
Australia	1 (7.1)
Asia	1 (7.1)
Study design	
Qualitative	12 (85.7)
Mixed Methods	2 (14.3)
Study population	
Sample size	
1–10	5 (35.7)
11–20	6 (42.9)
21–30	2 (14.3)
More than 30	1 (7.1)
Sample sex distribution	
Only female participants	4 (28.6)
More female participants	5 (35.7)
Equal number	2 (14.3)
More male participants	1 (7.1)
Not mentioned	2 (14.3)
Sample age range	
Child (0–13 years)	1 (7.1)
Child/Adolescent (0–17 years)	4 (28.6)
Adolescent (14–17 years)	3 (21.4)
Adolescent/Emerging adult (14–30)	1 (7.1)
Emerging adult (18–30)	4 (28.6)
Child/Adolescent/Emerging adult (0–30 years)	1 (7.1)
Parental mental illness	
Diverse disorders combined	6 (42.9)
Affective disorders	5 (35.7)
Schizophrenia	3 (21.4)

### Results of Quality Appraisal

The quality of the included studies was quite heterogeneous (as seen in Table 2). Four studies were assigned five stars, while one study did not meet any criterion, resulting in a score of 0%. Despite this, the study (Küçük Öztürk, 2022), a Lived Experience Narrative, was included due to the richness of coping instances mentioned. Overall, ten articles met at least 60% of all criteria. The qualitative studies that demonstrated lower quality lacked clear descriptions of their analysis method (Küçük Öztürk, 2022; Pölkki et al., 2005), had flaws in their analyses (Valiakalayil et al., 2004), or lacked a clear distinction between results and interpretation/discussion (Küçük Öztürk, 2022). The mixed-method study (Maybery et al., 2005), assessed as being of lower quality, failed to effectively integrate and interpret the different qualitative and quantitative study components and had deficits in both the qualitative and quantitative parts (e.g., lacking an adequate description of the analysis method or not describing the target population). Moreover, a clear research question was often missing, with only aims, purpose, or the focus of the respective study mentioned (Garley et al., 1997; Küçük Öztürk, 2022; Kuhn & Lenz, 2008; Maybery et al., 2005; Petrowski & Stein, 2016; Stelling et al., 2008; Trondsen, 2011; Valiakalayil et al., 2004).

**Table 2:** Quality appraisal (MMAT)

Qualitative studies								
Criterion	Clear research questions?	Data address research questions?	Qualitative approach appropriate?	Qualitative data collection method adequate?	Findings adequately derived?	Interpretation of results sufficiently substantiated?	Coherence between qualitative data sources, collection, analysis, and interpretation?	Overall quality of the study^a^
Brawer-Sherb et al	Yes	Yes	Yes	Yes	Yes	Yes	Yes	*****/100%
Garley et al	No	Unclear	Unclear	Unclear	Yes	Yes	Yes	***/60%
Kahl and Jungbauer	Yes	Yes	Yes	Yes	Yes	Yes	Yes	*****/100%
Küçük Öztürk	No	Unclear	Unclear	Unclear	No	No	Unclear	-/0%
Meadus and Johnson	No	Unclear	Unclear	Unclear	Yes	Yes	Yes	***/60%
Petrowski and Stein	No	Unclear	Unclear	Unclear	Yes	Yes	Yes	***/60%
Pölkki and Huupponen	Yes	Yes	Yes	Yes	Unclear	Unclear	Nein	**/40%
Stelling, Habers and Jungbauer	No	Unclear	Unclear	Unclear	Yes	Yes	Yes	***/60%
Trondsen	No	Unclear	Unclear	Unclear	Yes	Yes	Yes	***/60%
Valdez et al	Yes	Yes	Yes	Yes	Yes	Yes	Yes	*****/100%
Valiakalayil, Paulson and Tibbo	No	Unclear	Unclear	Unclear	Yes	Yes	No	**/40%
Van Parys et al	Yes	Yes	Yes	Yes	Yes	Yes	Yes	*****/100%

Mixed Method Studies								
Criterion	Clear research questions?	Data address research questions?	Adequate rationale for using mixed-method design?	Different study components effectively integrated?	Outputs of integration adequately interpreted?	Divergences between qualitative and quantitative results adequately addressed?	Different study components adhere to the quality criteria of each tradition of the methods involved?	Overall quality of the study
Kuhn and Lenz	No	Unclear	Yes	Yes	Yes	Yes	No	****/80%
Maybery et al	No	Unclear	Yes	No	No	Yes	No	**/40%

^a^ *20% of the criteria met; **40% of the criteria met; ***60% of the criteria met; ****80% of the criteria met; *****100% of the criteria met.

### Identified Families of Coping used by Children of Parents with a Mental Illness

The extracted coping instances were synthesized and assigned to the twelve families of coping as defined by Skinner and Wellborn (1994, p. 112). All families of coping and their related coping instances are presented here in detail. Table 3 provides an overview of all extracted coping instances, their assignment to the families of coping, their frequencies, and the associated studies.

**Table 3:** Identified families of coping used by offspring of parents with a mental illness

Family of coping	Coping instance	References
Self-reliance (11)^a^	• Working and studying hard to become successful • Positive attitude to life and pronounced self-esteem • Having time and space for yourself • Self-reliance/self-sufficiency • Positive self-instruction (reliance on oneself) • Optimistic attitude/ hope • Keeping emotions to oneself • Journaling • Quiet reflection • Expression of feelings toward parents, especially anger • Inner reflection	• Küçük • Stelling • Brawer-Sherb • Kahl • Trondsen •Valiakalayil • Van Parys
Support Seeking (29)	• Support seeking (conversations with peers about maternal illness, or with toy, pet, imaginary friend) • Family prayer • Support by extended family, siblings, fathers • Sharing feelings • Talk with the ill parent directly • Social support (siblings and friends) • School counselor • Take advantage of social support in neutral areas (e.g., with schoolwork, household matters) • Conversations with mother about mental illness • Receiving care from siblings • Support from peers • Social (emotional) support by friends and family (siblings), mentors, romantic partners, their mother • Social support by friends • Talking about the illness • Social support by parents or siblings • Support and help by friends and partners • Support by ill parent/healthy parent, siblings, grandparents, aunts, and uncles • Social support by siblings • Social support by friends (going to their house—physical distance) • Talking to others • Talking with friends • Support seeking (from professionals) • Talk to others • Meet friends • Talking to a sibling, friend, or another family member • Prayer • Forging of social support (family: grandparents, but also parents sometimes, siblings, friends, teachers, mental health workers) • Go to therapy (for self-reflective processes) • Dialogue with significant others	• Valdez • Pölkki • Küçük • Kuhn • Petrowski • Stelling • Brawer-Sherb • Garley • Kahl • Maybery • Meadus • Trondsen • Valiakalayil • Van Parys
Problem Solving (6)	• Providing mothers with space (physical and emotional) • Own problem-solving strategies / try to figure it out myself • Increased sensitivity to parents’ moods/recognize early warning signs of the disease • Solving problems alone • Emotional support for father • Actively and patiently search for a solution • Controlling the situation (explaining that hallucinations can’t be true to ill parent) • Reassuring parents	• Valdez • Küçük • Kuhn • Petrowski • Stelling • Kahl • Van Parys
Information Seeking (5)	• Reading books about mental illness • Conversations with mother about mental illness • Quest for information (allows for detecting signs of illness) • Seeking information about the parental mental disorder • More information gathering	• Küçük • Petrowski • Garley • Meadus • Trondsen • Van Parys
Accommodation (22)	• Distractions outside the home (sports, biking, movies, time with friends) • Allowing time to see what would happen • Family time (movies, exercising, playing board games) • Importance of learning and inner growth • Pleasant hobby • Studying lessons • Playing volleyball and going trips • Avoid thinking about mental illness (listening to music, reading a book, dreaming, and trying to be happy—form of meditation) • Social activities • Hobbies • Activities for short-term relief and relaxation (e.g., relaxing, listening to music, sport, reading) • Diverting attention (reading, sports, TV) • Distraction (cognitively: fantasy; play with pet or toys) • Acceptance of the parental mental illness • Hobbies (function: meet friends and receive credit for achievements) • Humor • Thinking positively and trying to see the illness as just like any other illness • Focus on positive aspects of their experience (e.g., being grateful) • Meet friends • Work out • Differentiation between parent and illness • Ability to put things into perspective	• Valdez • Pölkki • Küçük • Stelling • Garley • Kahl • Meadus • Trondsen • Van Parys
Negotiation (5)	• Regulate amount of personal sharing/type of information • Regulate communication to not upset or stress ill parent • Adjust own behavior • Balancing what can be said/shared of own experiences and emotions • Balancing belonging and differentiation with parent	• Petrowski • Trondsen • Van Parys
Delegation (0)	/	/
Isolation (13)	• Negation of the disorder/strain • Limit amount of contact/avoid conflict • Physical and/or emotional distancing • Close herself off emotionally • Denial • Avoiding to talk about the illness • Avoidance of the topic of mental illness outside of the family • Keep to oneself/ rarely ask for help • Stop social contact • Detachment • Keeping emotions to oneself • Keeping illness a secret • Silencing their own worries and distress/hiding anger	• Kuhn • Petrowski • Brawer-Sherb • Garley • Kahl • Maybery • Meadus • Trondsen • Valiakalayil • Van Parys
Helplessness (3)	• Close herself off emotionally • Sadness, separation anxiety, excessive demand, helplessness and sometimes even feelings of guilt • Feelings of sadness, loss, fear and frustration	• Brawer-Sherb • Kahl • Valiakalayil
Escape (13)	• Working and studying hard to become successful • Avoidance of thinking about the disorder/defense • Internal distance • Avoidance of the topic • Trivialization • Passive-avoidant reactions (e.g., distancing until situation calms down) • Withdrawal (with more independence) • (School) withdrawal • Social support by friends (going to their house—physical distance) • Avoid difficult situations • Physical and emotional distance/time-out (retreat to a special place/outdoors) • Physical and emotional distance by moving away • Distancing (physically and psychologically)	• Küçük • Kuhn • Stelling • Kahl • Maybery • Trondsen • Van Parys
Submission (2)	• Deny emotional needs • Continuous thoughts about experienced situations and worries	• Pölkki • Kahl
Opposition (6)	• Aggressive behavior to regulate emotions • Aggressive behavior • Anger • Anger and resentment • In adolescence/young adulthood: expression of feelings toward parents, especially anger • Anger and resentment	• Kuhn • Kahl • Meadus • Valiakalayil • Van Parys
Parentification/Role reversal (14)	• Household chores • Assuming responsibility for the parents • Role reversal/parentification • Giving care for siblings • Role reversals/loyalty toward their parents • Taking on extra roles (parentification) • Role reversal • Taking responsibility/role reversal • Doing housework • Taking care of younger siblings • Role reversal • Role reversal (supporting parent emotionally) • Go to therapy (for self-reflective processes)	• Garley • Maybery • Meadus • Trondsen • Valiakalayil

^*a*^*Numbers in brackets show how many codes were identified*

### Self-Reliance

Serving the adaptive function of coordinating reliance on social resources (relatedness), the family of coping called self-reliance involves “active attempts at self-care and regulation of distress” (Skinner & Zimmer-Gembeck, 2016, p. 39) through self-soothing, self-encouragement, emotional control, relaxation, or authentic emotional expression.

In the present review, eleven coping instances were assigned to this category. In some articles, children, adolescents, or emerging adult children mentioned dealing with the parental mental illness by having time and space for themselves (Stelling et al., 2008), by relying on themselves (Brawer-Sherb et al., 2022; Kahl & Jungbauer, 2014), by journaling (Valiakalayil et al., 2004), or through quiet inner reflection (Valiakalayil et al., 2004; Van Parys et al., 2015). The following quotes illustrate the reliance of children, adolescents, and emerging adult children of parents with a mental illness on themselves:

- “I first try to resolve the problem by myself” (Kahl & Jungbauer, 2014, p. 189)
- “I just try to live my own life and focus on myself, I’m in grad school and I’m really busy. I just try to get very involved with school and not worry about the texts she sends me, they are not anything urgent or important” (Brawer-Sherb et al., 2022, p. 760)

One adult child spoke about how working and studying hard to become successful helped her focus on herself (Küçük Öztürk, 2022). Others coped with the parental mental disorder through authentic emotional expression, by feeling emotional relief after expressing anger toward their parents (Valdez et al., 2019; Van Parys et al., 2015) or by controlling their emotions (Trondsen, 2011). In some studies, participants relied on their positive attitude to life, hope, and pronounced self-esteem to help them cope with the everyday challenges of living with a parent with a mental illness (Kahl & Jungbauer, 2014; Stelling et al., 2008).

### Support Seeking

Like self-reliance, support seeking serves the adaptive function of relatedness. However, unlike self-reliance, it is directed toward the context rather than the self. Support seeking encompasses all reactions intended to “signal and reach the support provider” (Skinner & Zimmer-Gembeck, 2016, p. 39) like seeking comfort, praying, or by imaging what others would say.

In the present review, support seeking was the most frequently coded family of coping, with a total of 29 instances. The majority of these instances described seeking social support from family, especially siblings, extended family like grandparents, aunts, and uncles, and from friends (Brawer-Sherb et al., 2022; Garley et al., 1997; Kahl & Jungbauer, 2014; Kuhn & Lenz, 2008; Maybery et al., 2005; Petrowski & Stein, 2016; Pölkki et al., 2005; Stelling et al., 2008; Trondsen, 2011; Valdez et al., 2019; Van Parys et al., 2015).

Others explicitly described how talking with someone helped them, regardless of whether the conversation was about the illness (Garley et al., 1997; Meadus & Johnson, 2000; Petrowski & Stein, 2016; Trondsen, 2011; Valdez et al., 2019; Valiakalayil et al., 2004; Van Parys et al., 2015). One child even spoke of how talking with her pet made her feel better: “When I was sad in the morning. I used to get my bunny out of his cage, and I used to put it on the couch and talk to it and kiss it... he’s cuddly and soft. [I talked to it] because it doesn’t talk, it can keep my secrets” (Valdez et al., 2019, p. 995).

Some children and adolescents sought support from professionals (Küçük Öztürk, 2022; Trondsen, 2011; Van Parys et al., 2015) although this was sometimes experienced as challenging: “It’s like I don’t want to go back to the real world. It’s tiring to sit over there and talk about thoughts and experiences and painful things, and then have to go out and smile and pretend I’m happy again, like nothing has happened” (Trondsen, 2011, p. 182).

In two articles, prayer was mentioned as a support-seeking strategy (Valdez et al., 2019; Valiakalayil et al., 2004).

### Problem Solving

Problem solving serves the adaptive function of coordinating actions and contingencies in the environment. It involves action tendencies “characterized by active attempts to produce effects, the emotion of determination, and an attentional focus during transactions on discovering how to produce desired outcomes” (Skinner & Zimmer-Gembeck, 2016, p. 37). In the present review, problem solving was coded six times. Participants described engaging in problem-solving behaviors, such as independently seeking solutions to issues and attempting to control the situation (Kahl & Jungbauer (2014), Küçük Öztürk (2022), Kuhn and Lenz (2008), and Valdez et al. (2019)). A poignant example is provided by Stelling et al. (2008), who quote a 20 year-old son of a mother with mental illness: “So, if there’s a problem, you first try to solve it yourself somehow. That you don’t give up straight away, but that there is a solution for every problem. And that you can tackle it yourself.” (Stelling et al., 2008, p. 765). This quote exemplifies the determination and proactive approach typical of problem-solving coping strategies among children of parents with a mental illness. One study even shows how participants preventively deal with their parent’s mental illness by having an increased sensitivity to their parents’ moods and recognizing early warning signs of the disease (Kuhn & Lenz, 2008).

### Information Seeking

Information seeking refers to efforts to find out more about a situation, “including its course, causes, consequences, and meanings, as well as learning about strategies for intervention and remediation” (Skinner & Zimmer-Gembeck, 2016, p. 37). This search for information can involve social partners (e.g., seeking advice, consulting experts) and other methods (e. g. reading information materials, taking notes on one’s health). Like the coping family of problem solving, information seeking serves the basic human need of feeling competent. In this review, information seeking was coded five times. Participants used both social and impersonal methods to gather information. They reported reading books about mental illness (Küçük Öztürk, 2022), asking their family doctor for information (Meadus & Johnson, 2000), volunteering at a self-help organization for individuals diagnosed with a mood disorder (Meadus & Johnson, 2000), or asking the healthy parent for more information (Garley et al., 1997). Only in one study was direct communication with the ill parent reported as a coping strategy. A young adult daughter of a mother diagnosed with major depression, who was also experiencing depression herself, stated:

“When I started to show signs [of depression], is when my mother first sat down and talked to me….And she told me everything about my grandma and her and everything that was going on, and so that’s how I learned about her suffering with it. And she told me that she still suffers with it and that made me feel better as I was going through it.” (Petrowski & Stein, 2016).

### Accommodation

Besides relatedness and competence, human beings have a basic need for autonomy. Coping families based on this need aim to coordinate preferences and available options, either directed at oneself (accommodation and submission) or the context (negotiation or opposition). Accommodation, also known as secondary control coping, includes “processes by which people flexibly adapt their preferences to the options available in stressful situations” (Skinner & Zimmer-Gembeck, 2016, p. 40). This can involve distraction as well as positive cognitive restructuring. In the present review, accommodation, closely following support seeking, was one of the most frequently coded coping families, with a total of 22 instances. More than half of these instances were related to distraction through pleasant activities, which could be solitary or social. Participants reported meeting friends, doing sport, listening to music, reading, or spending time with family or pets to relax and distract themselves (Garley et al., 1997; Kahl & Jungbauer, 2014; Küçük Öztürk, 2022; Pölkki et al., 2005; Stelling et al., 2008; Trondsen, 2011; Valdez et al., 2019). One child of parents with a mental illness explained how working and studying hard helped her cope with the parental mental illness (Küçük Öztürk, 2022). The second aspect of accommodation, positive cognitive restructuring, was coded eight times. Participants coped by accepting the situation, allowing “time to see what would happen” (Valdez et al., 2019, p. 996), using humor to manage their circumstances (Meadus & Johnson, 2000, p. 387), emphasizing the “importance of learning and inner growth” (Pölkki et al., 2005, p. 160), or focusing on the positive aspects of their experience (Meadus & Johnson, 2000). The perceived personal growth and development is well illustrated by the following quote:

“I feel like I have a different kind of wisdom, like I’m more mature than my peers. I feel they can’t possibly understand because after my father’s depression, I really started to think a lot about the meaning of life. (. . .) I also listen to lyrics that are very profound and then I think about them (. . .) and write up some things myself.” (Van Parys et al., 2015, p. 530)

### Negotiation

The coping family of negotiation serves the underlying function of autonomy and is directed at the context. Negotiation involves “active attempts to work out a compromise between the priorities of the individual and the constraints of the situation” (Skinner & Zimmer-Gembeck, 2016, p. 40). It encompasses behaviors such as setting priorities, proposing compromises, persuasion, constructive resistance, defending one’s goals, and reaching agreements. In the present review, negotiation was coded five times. Participants reported adjusting their behavior to the specific circumstances of living with a parent with a mental disorder. This included regulating the amount of personal sharing and type of information to avoid upsetting or stressing their ill parent (Petrowski & Stein, 2016; Van Parys et al., 2015) or trying to avoid actions that could disturb the parent (Trondsen, 2011). One example is provided by a young woman with a mother who is affected by mental illness: “I had to watch what I’ve said more, because if I said something that really upset her, then it would just throw her into a downward spiral, and I didn’t want to see that.” (Petrowski & Stein, 2016, p. 2879).

### Delegation

Delegation refers to excessive “over-reliance on other people in dealing with stressful situations” (Skinner & Zimmer-Gembeck, 2016, p. 39). It belongs to the group of coping families related to the need for relatedness, where the adaptive function involves coordinating reliance and available social resources. However, unlike self-reliance and support seeking, the individual’s distress in delegation is high, surpassing their regulatory capacities. As a result, delegation is considered a maladaptive form of coping with stress (Skinner et al., 2003). Examples of this behavior include dependence, inappropriate help seeking, whining, and self-pity. In the present review, no instances of coping were assigned to delegation.

### Isolation

When the need of relatedness is threatened and individuals do not perceive support seeking as effective (or lack the means to use this coping strategy), they might turn to coping strategies from the family of isolation. Isolation involves “actions aimed at withdrawing or staying away from other people, either physically or psychologically, for example, by preventing other people from knowing about a stressful situation or its emotional effects” (Skinner & Zimmer-Gembeck, 2016, p. 39). In this review, coping instances were assigned to social isolation 13 times. Participants reported both physical isolation (coded three times) and emotional isolation (coded ten times). They limited the amount of contact (Petrowski & Stein, 2016), distanced themselves physically – even moving abroad (Brawer-Sherb et al., 2022) – or ceased social contact altogether (Maybery et al., 2005). A poignant example comes from a child whose mother was unwell: “my dog is the closest thing I have to human contact for days when mum is in hospital” (Maybery et al., 2005, p. 6).

In addition to physical isolation, many coping instances revealed forms of emotional isolation. Offspring of parents with a mental illness often negated the disorder or strain (Garley et al., 1997; Kuhn & Lenz, 2008), closed themselves off emotionally (Brawer-Sherb et al., 2022; Meadus & Johnson, 2000; Trondsen, 2011), or avoided discussing the illness (Garley et al., 1997; Valiakalayil et al., 2004). One child affected by parental schizophrenia stated: “I never dared to say anything because I always found it silly” (Kahl & Jungbauer, 2014, p. 188). Keeping the illness a secret and shutting off own emotions sometimes meant silencing their worries and distress, as well as hiding anger. One young adult with a mother suffering from depression recalled a situation with her mother: “I always reassured her ‘no I’m alright, I’m alright’,... whereas I probably was not alright at all at that moment.” (Van Parys et al., 2015, p. 528).

### Helplessness

Helplessness refers to the “dejected withdrawal of active attempts to change the situation accompanied by discouragement and resignation” (Zimmer-Gembeck & Skinner, 2016, p. 38). This coping family includes behaviors such as giving up, relinquishing control, and experiencing feelings of pessimism and passivity. In the present review, helplessness was coded three times. Participants reported feelings of sadness, loss, fear, and frustration (Valiakalayil et al., 2004), as well as experiences of separation anxiety, helplessness, and sometimes even guilt (Kahl & Jungbauer, 2014), or closed themselves off and resigned:

“I feel like when I talk to her, her affect is so flat. When she takes her medication she’s so out of it. I’ll be out with my friends and I’ll call my mom but I know she’s going to be asleep so she doesn’t know what I’m doing. I don’t talk to her, and I get frustrated so I don’t even want to tell her things. It’s just not even worth it or she’s just not going to remember what I said to her. I’ve always been close with my mom and that’s been really hard because I’ve had to, well, I haven’t had to back away, but I think I just have subconsciously, so it’s just not the same way it used to be.” (Brawer-Sherb et al., 2022, p. 759).

### Escape

Similar to helplessness, escape represents a coping family related to competence. When distress levels are high, individuals may choose to escape a stressful environment, either behaviorally by physically leaving the situation, or cognitively through cognitive avoidance, denial, or wishful thinking. In this review, escape was coded 14 times. Participants reported physically distancing themselves by going to their friend’s house (Maybery et al., 2005), withdrawing from school (Maybery et al., 2005), retreating to a special place or the outdoors (Trondsen, 2011), avoiding difficult situations (Trondsen, 2011) or moving away entirely (Trondsen, 2011). The latter is illustrated by an adolescent with a mother with a mental illness: “What needed to happen was me moving out, you see, so that she could begin to take proper care of herself. This is something she does now. But she wanted to have me there, like a sort of nurse.” (Trondsen, 2011, p. 183). In one study, working and studying hard to escape the situation was also mentioned (Küçük Öztürk, 2022). Participants also reported cognitive methods of escape, such as avoiding thoughts about the disorder (Kuhn & Lenz, 2008), avoiding the topic, or trivializing it (Kahl & Jungbauer, 2014; Stelling et al., 2008). The health-promoting effect of distancing is illustrated by the following quote from a young adult affected by parental depression:

“I can distance myself from that [his father’s depression]. I’ve been abroad for some time, that changes a lot. [. . .] So what you need is an ‘outsider perspective’, as long as you have that critical perspective of an outsider. . . You have to find a way to deal with it in your daily life, so that it does not interfere too much with your own life, and your will, and your routine, and your social life.” (Van Parys et al., 2015, p. 531).

### Submission

When the basic human need for autonomy is threatened, and stress levels surpass an individual’s coping resources, they may resort to strategies form the coping family of submission. Submission is described as “a grudging resigned surrender to stressful events” (Zimmer-Gembeck & Skinner, 2016, p. 40) and includes lower-order ways of coping such as rumination, intrusive thoughts, negative thinking, catastrophizing, anxiety amplification, self-blame, or fear. Submission is often viewed as an involuntary engagement in stress responses (Zimmer-Gembeck & Skinner, 2016, p. 40). In the present review, only two coping instances were assigned to submission (Kahl & Jungbauer, 2014; Pölkki et al., 2005). The following quote is illustrative of the submissive coping reactions that offspring of parents with a mental illness might exhibit: “Sometimes, when I did something wrong, I think about it, even if it’s already long ago.” (Kahl & Jungbauer, 2014, p. 189).

### Opposition

Like submission, opposition is a coping family used in situations where autonomy is threatened. When distress levels are high and negotiation is not a viable option, individuals may resort to strategies aimed at attacking the perceived source of stress. Opposition encompasses lower-order ways of coping such as “aggression, projection, reactance, confrontation, defiance, revenge, discharge, venting, and blaming others” (Zimmer-Gembeck & Skinner, 2016, p. 41). In the present review, six instances were assigned to the family of coping of opposition. The included studies reported aggressive behavior (Kahl & Jungbauer, 2014; Kuhn & Lenz, 2008), as well as expressions of anger or resentment (Meadus & Johnson, 2000; Valiakalayil et al., 2004; Van Parys et al., 2015). One young adult described how she released her emotions in front of her parents:

“At that moment I poured out so many things that I had been bottling up before. I cannot remember that I had ever been so mad at my parents, and I expressed my anger towards them. [. . .] I think I used to keep it for myself. [. . .] I felt very relieved.” (Van Parys et al., 2015, p. 532).

### Role Reversal

Role reversal does not belong to the twelve families of coping outlined by Skinner and colleagues (2003). However, some coping instances extracted from the included studies could not be assigned to this system of coping families. In many of the studies, participants reported assuming responsibilities and roles typically held by parents. These behaviors must be distinguished from problem-solving strategies due to the specific nature of role reversal and parentification. Therefore, we propose a new family of coping: role reversal. In this review, role reversal was coded 14 times, making it one of the most widely used coping family after support seeking and accommodation. One participant explicitly described her experience of reversed roles with her father: “Sometimes it’s more like I’m the one who is the parent, and he is the child. I feel like he’s my responsibility, and that I have to take care of him.” (Trondsen, 2011, p. 181). Coded instances of role reversal demonstrated both emotional and instrumental parentification. Offspring of parents with a mental illness reported assuming responsibilities for their parents (Kuhn & Lenz, 2008; Trondsen, 2011), providing emotional support (Petrowski & Stein, 2016; Van Parys et al., 2015), caring for their siblings (Petrowski & Stein, 2016; Trondsen, 2011), or managing household task (Küçük Öztürk, 2022; Trondsen, 2011). The following quote illustrates the emotional parentification an adolescent experienced:

“My dad likes me to be there because I’m the only person who listens to him and understands what he’s going through . . . other people . . . don’t understand . . . So I’m just there listening, give my opinion the best I can, and according to my age, what I can do . . . There’s not much I can do.” (Garley et al., 1997, p. 101).

### Coping Instances Relating to Challenge and Threat

Based on the motivational theory of coping (Skinner & Wellborn, 1994), the extracted coping instances were categorized into families of coping that relate to either challenge or threat. Families of coping associated with challenge are characterized by a manageable level of distress, allowing individuals to address stressors in adaptive ways. According to Skinner and colleagues (2003), six families of coping represent adaptive strategies: self-reliance, support seeking, problem solving, information seeking, accommodation, and negotiation. In contrast, families of coping associated with threat involve distress levels that exceed an individual’s regulatory capacity, leading to maladaptive coping. These six maladaptive families of coping include delegation, isolation, helplessness, escape, submission, and opposition (Skinner et al., 2003).

In the present review, 78 coping instances were classified as adaptive, while 37 were categorized as maladaptive. These figures exclude coping instances assigned to the newly created category of role reversal. Among adaptive coping strategies, the most frequently reported were support seeking and accommodation. Conversely, among maladaptive strategies, offspring of parents with a mental illness most reported coping instances related to escape and social isolation.

## Discussion

Coping is understood as regulating oneself under psychological distress. In the transgenerational transmission of psychopathology from the parent to the child generation, it is identified as one important mediator. However, a systematic review of qualitative literature regarding the coping of children, adolescents, and young adult children of parents with a mental illness has not yet been conducted. The present review therefore represents the first synthesis of qualitative literature examining the coping of children, adolescents, and emerging adult children of parents with a mental illness.

Regarding research question 1 and 2, on what types of coping instances are reported by this population and how they can be organized into Skinner’s higher-order families of coping, we found that they most frequently employed support seeking and accommodative strategies to cope with their parents’ mental illness. The coping families of escape and social isolation were also reported frequently, followed by self-reliant strategies. Fewer instances of coping were categorized under other families of coping. However, we identified a new family of coping that is not part of Skinner and colleagues’ (2003) twelve families of coping. This newly proposed higher-order category, termed role reversal, encompasses behaviors of emotional and instrumental parentification and was observed in nearly 65% of the included studies.

Comparing these findings with results from normative samples reveals both similarities and differences. In a review including 62 studies, Skinner and Zimmer-Gembeck (2016) examined age-graded changes in the coping strategies of children and adolescents faced with various stressors. In both reviews, participants frequently reported using support seeking and distraction (a form of accommodative strategy). Similarly, escape was used to a relatively high degree in both contexts. The frequent use of support seeking, distraction, and escape suggests these coping families may be universally employed by children and adolescents, regardless of the specific stressor. However, the coping family of problem solving, which was heavily used in the review of normative samples (Skinner & Zimmer-Gembeck, 2016), was far less frequently employed by offspring of parents with a mental illness. In contrast, these individuals relied more heavily on behaviors related to role reversal. While some of these behaviors might resemble problem solving, their distinct nature warranted framing them as a new family of coping in this review. The key characteristic of role reversal is the assumption of developmentally inappropriate adult roles and parentification, which is not observed in problem-solving coping strategies within normative samples. Additionally, offspring of parents with a mental illness used more coping strategies linked to social isolation than those in normative samples. This withdrawal from social interaction may be due to experiences or fears of stigma-by-association (Dobener et al., 2022; Reupert et al., 2021). It may also be due to living in familial and societal environments where parental mental illness is considered a taboo (Yamamoto & Keogh, 2018).

The evidence from reviews on the coping of children, adolescents, and young adults facing specific stressors also offers valuable insights when compared with our findings. Thulstrup and Carlsson (2017), in their review of coping strategies among children with incarcerated parents, identified a similar pattern. Notably, in both reviews, problem solving was not identified as a prevalent strategy. Both groups—children of incarcerated parents and offspring of parents with a mental illness—face uncontrollable stressors, which may explain their preference for emotion-oriented or avoidant strategies over active, problem-focused ones (Forsythe & Compas, 1987). Moreover, parents influence all phases of their children’s coping process, from providing the context for potential stressful events to coaching and modeling appraisal reactions and coping behaviors (Power, 2004). An empirical study by Liga and colleagues (2020) investigated coping socialization and found direct positive relationships between the coping strategies used by fathers and mothers and the use of the same strategies by adolescents. Coping socialization also occurs in families with parental mental illness. Cognitive deficits, particularly problems with executive functioning (e.g., problem solving), are common in most mental disorders (Trivedi, 2006). Significant deficits in problem solving have been long recognized in individuals with depression (Nezu, 1987) and schizophrenia (Mihaljević-Peleš et al., 2019). In the current review, affective disorders (35.7% of all included studies) and schizophrenia (21.4% of all included studies) were the most frequently reported parental mental disorders. Consequently, parental deficits in problem solving might have resulted in low levels of this coping strategy among the group of interest in this review due to a lack of parental social modeling.

Coping strategies involving role reversal were identified in a review on children’s coping with interparental conflict (Kerig, 2001). Similar to the context of parental mental illness, interparental conflict may impair parents’ ability to fulfill their parental roles, leading children to take on developmentally inappropriate responsibilities. Interestingly, while role reversal emerged as a prevalent coping strategy, the family of coping related to delegation was not coded at all. Delegation involves relinquishing responsibility and depending on others, contrasting sharply with the behaviors associated with role reversal, where parentified children take on their parents’ responsibilities. The absence of delegation in the present results suggests that, for offspring of parents with a mental illness, relying on others is not a viable coping option. This could indicate a lack of available support systems or resources, further emphasizing the unique challenges faced by this population in managing their circumstances. Furthermore, (feared) stigma-by-association (Dobener, 2022) might also discourage the offspring of parents with a mental illness from using delegation as a coping strategy.

Regarding research question 3, on how reported coping instances relate to challenge and threat, we found that according to the hierarchical conceptualization of coping by Skinner and colleagues (2003), most instances were classified as adaptive. While this model provides a powerful and useful framework for organizing coping strategies, certain weaknesses and limitations became evident throughout the review. Firstly, Skinner and colleagues (Skinner et al., 2003, p. 243) proposed the mutual exclusivity of coping families, suggesting that each coping instance belongs to only one family. However, our findings do not fully support this assumption, as some instances showed how a single coping instance can span multiple families and functions. Therefore, the mutual exclusivity or potential overlap between coping families deserves more in-depth investigation. Secondly, several studies included in the review (Küçük Öztürk, 2022; Meadus & Johnson, 2000; Petrowski & Stein, 2016) reported coping instances where individuals coped by choosing a helping profession or supporting others affected by mental illness, such as through involvement in mental health organizations. These instances could be seen either as a long-term coping strategy or as an outcome of growing up with a parent with a mental disorder and were not coded in the present review due to the focus on situational coping strategies. The model by Skinner and colleagues does not account for such phenomena, which may represent a significant form of coping for some offspring. This idea is also supported by existing literature suggesting that children of parents with a mental illness often gravitate toward helping professions (Bucco et al., 2025). Thirdly, the general categorization of coping families into adaptive and maladaptive categories does not adequately reflect the unique living conditions of children, adolescents, and emerging adult children of parents with a mental illness. For example, physical and emotional distancing was coded as escape, which is generally seen as maladaptive. However, in the specific context of parental mental illness, distancing might be a highly reasonable and protective response to a stressful or even potentially harmful situation. This suggests that coping behaviors, typically labeled as maladaptive, might serve an important function under certain circumstances, particularly in high-risk environments. Similarly, when children attempt to actively change their situation, this is coded as problem solving. While these children may be engaging in an active, goal-oriented strategy, it often fails due to the nature of the stressor, which may be beyond their control. For the complex and uncontrollable stressors due to parental mental illness, the classification into adaptive and maladaptive strategies must be approached with caution. Lastly, the coping system proposed by Skinner and colleagues (2003) fails to encompass all coping strategies employed by offspring of parents with a mental illness. Notably, instances of parentification among these youth led to the establishment of a new coping family called role reversal. This new classification underscores the significant emotional and instrumental responsibilities these children take on, often stepping into parental roles.

### Model of Coping of COPMI

To address the unique context of offspring of parents with mental illness, a new model of coping is proposed here. This model aims to address our criticisms of Skinner and colleagues’ hierarchical conceptualization of coping (2003) by incorporating several key modifications: The model adds role reversal as a distinct family of coping, acknowledging the significant emotional and instrumental responsibilities assumed by children in these situations. Moreover, instead of categorizing coping families based on their potential adaptiveness, the model recognizes the complexity of coping responses and their situational appropriateness, emphasizing that what may be adaptive in one context can be maladaptive in another.

**Fig. 3:**
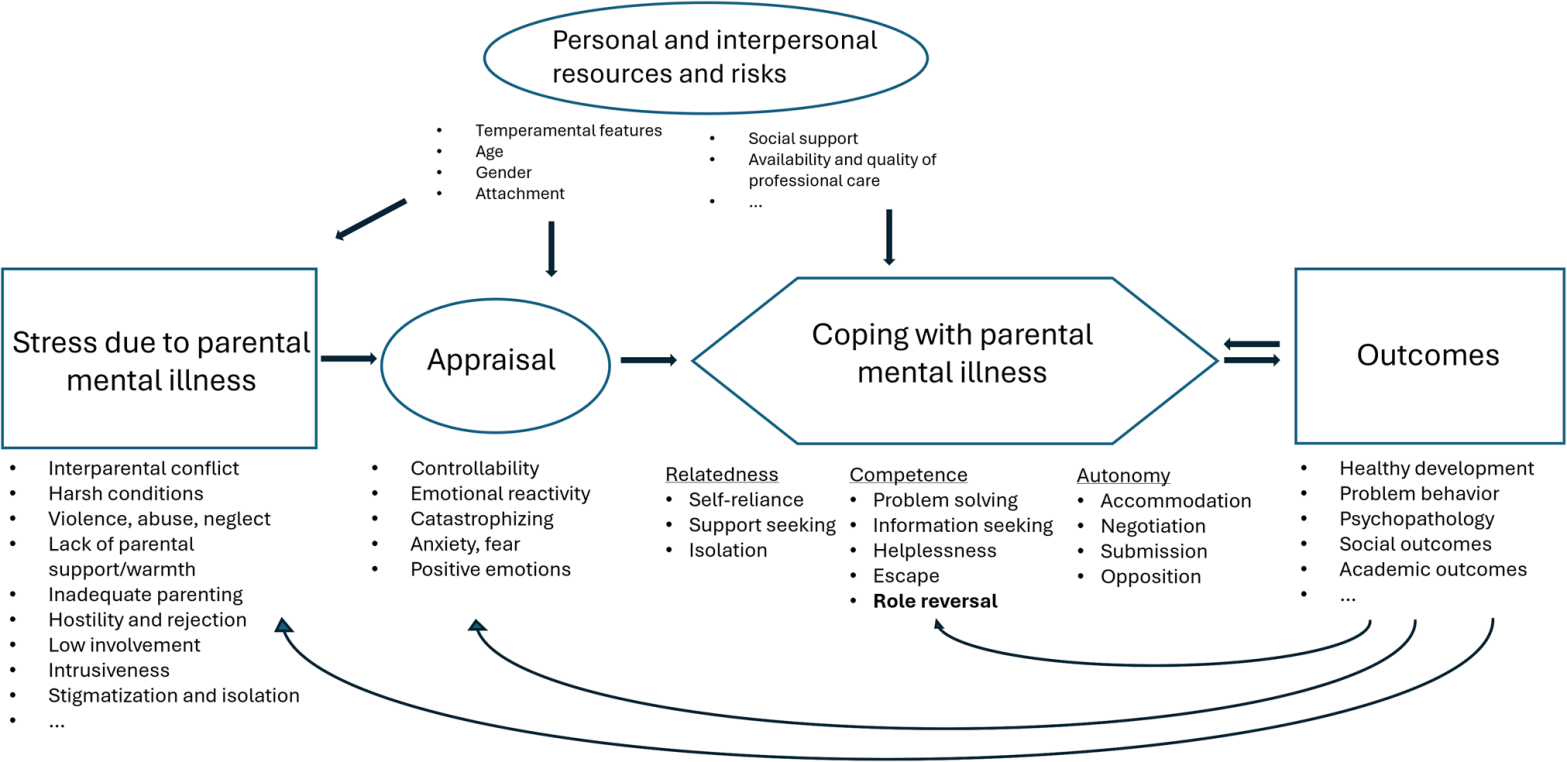
Model of Coping of COPMI

The proposed model is situated within the framework of the Transactional Theory of Stress and Coping (Lazarus & Folkman, 1984) and is grounded in the model of academic coping by Skinner and Saxton (2019). It incorporates the twelve families of coping identified by Skinner and Wellborn (1994) along with the findings from the present review. Furthermore, it draws on elements from the developmental model of transgenerational transmission of psychopathology by Hosman and colleagues (2009). The model of coping of COPMI is designed as a combined process- and content model. It illustrates the progression from initial stress to appraisal, followed by coping strategies, potential outcomes, and feedback loops. Simultaneously, it categorizes coping strategies into families, thereby structuring the content of coping in a way that reflects the complexities and nuances of the experiences faced by children, adolescents, and young adults of parents with a mental illness (see Fig. 3).

The proposed model for understanding coping in children of parents with a mental illness is structured around several key components that reflect the complexities of their experiences. The model identifies stressors stemming from parental mental illness, which can manifest as specific stressors that occur independently or concurrently. This categorization facilitates an exploration of coping strategies for particular stressors associated with various parental mental disorders. A transdiagnostic approach is emphasized, recognizing that many mental disorders exhibit overlapping symptoms. Appraisal serves as a mediator in the coping process, determining how children perceive and interpret the stressors they encounter. Coping is organized into the coping families identified in the present review, with the addition of the newly established family of role reversal. This classification allows for a nuanced understanding of how children navigate their unique challenges. The outcomes of the coping process can be divided into healthy development, psychopathology, social outcomes, and academic and professional outcomes (including career choices). These outcomes can affect various stages of the coping process, including the emergence of stressors, appraisal, and the selection of coping strategies. Both personal and interpersonal factors play crucial roles in shaping the coping experience: Interpersonal resources and risks encompass protective and risk factors in the child’s social environment, such as family support, community resources, and stigma. Personal resources and risks are individual characteristics, including temperamental traits, age, and gender that can influence how children respond to stressors and cope.

The proposed model of coping for offspring of parents with a mental illness presents a flexible framework that can be integrated with other theoretical models to enhance understanding of the unique challenges faced by this population. For example, the model by Blake-Holmes (2023) that explored the emergence of parentification through narratives of adult children of parents with a mental illness, can be incorporated into the present framework. By detailing how parentification occurs, it enriches the understanding of role reversal within the COPMI model, offering a more nuanced perspective on this specific coping strategy. The model is also adaptable to other contexts, such as interparental conflict. Like parental mental illness, interparental conflict can disrupt parental roles and responsibilities, potentially leading to comparable coping responses in children. Similarly, the model could accommodate the unique stressors associated with parental incarceration. The dynamics of parental absence and the resulting coping mechanisms can be explored within this framework, highlighting the potential for role reversal and other adaptive or maladaptive strategies. The underlying assumption that any stressor compromising a parent’s ability to fulfill their role could be addressed by the model, demonstrating its broader applicability. Future research can validate this assumption by examining various stressors and their impact on coping strategies among affected children.

### Strengths and Limitations

The present work represents the first systematic review and meta-synthesis focused on the coping strategies of offspring of parents with a mental illness, synthesizing findings across different age groups. However, some limitations of the present review process must be acknowledged. First, the number of included studies was limited (k = 14), which restricts the generalizability and external validity of the findings. Furthermore, the quality of the included studies varied significantly, contributing to heterogeneity in the findings. Diagnoses of the affected parent were not formally assessed in all papers; in some cases, the evaluation was based on self-reports of the parent (Maybery et al., 2005) or on third-party report by their children (Brawer-Sherb et al., 2022; Küçük Öztürk, 2022; Petrowski & Stein, 2016; Pölkki et al., 2005; Trondsen, 2011; Van Parys et al., 2015). The use of self-reports from parents regarding their mental health diagnoses raises concerns about accuracy and reliability. Parents may underreport or misreport their conditions due to stigma or lack of insight. While children can provide valuable perspectives, their assessments may be influenced by their own experiences and perceptions, leading to potential biases in evaluating the severity of the parental mental illness. The potential biases related to the assessment of parental mental illness can affect the conclusions drawn about the coping strategies employed by the children, adolescents, and young adults.

Despite these limitations, this review is the first one to highlight the necessity of adapting traditional conceptualizations and models of coping, which primarily address normative samples, to better reflect the unique circumstances faced by children, adolescents and young adults of parents with a mental illness. Accordingly, a new model of coping of COPMI has been proposed that integrates role reversal as a distinct coping family.

### Implications for Research and Practice

The present findings highlight that offspring of parents affected by mental illness predominantly utilize support-seeking and accommodative coping strategies. Therefore, interventions should aim to reinforce these adaptive strategies. Additionally, particular attention should be given to the phenomenon of role reversal. Professionals need to be trained to recognize early signs of parentification, and parents facing mental health challenges should be educated about its implications for their children. Supporting affected children in understanding their roles as children, while reinforcing their ability to identify and meet their own needs without shame, is essential. Furthermore, family-wide programs, such as the Family Talk Intervention by Beardslee and colleagues (2003), along with external support services, could alleviate strain and provide relief for these children. Strengthening social networks and facilitating peer support groups can also help reduce isolation and foster a sense of normality.

The findings further indicate that offspring of parents with a mental illness employ distinct coping strategies compared to normative samples. As a result, measurement tools for coping should be adapted to account for the specific context of a parental mental illness, particularly by assessments of parentification.

Furthermore, the proposed model of coping of COPMI invites future researchers to explore the relationships between specific stressors associated with a parental mental illness and various outcomes. Investigating how particular coping strategies impact these relationships will deepen our understanding of the coping process for offspring of parents with a mental illness. To validate the model’s adaptability, empirical studies could explore how children cope in different challenging contexts (e.g., interparental conflict, parental incarceration) and identify commonalities and differences in coping strategies, as well as their associations to health outcomes. The newly created coping family, role reversal, also warrants further examination. Understanding the developmental phases of parentification might help to identify preventive measures that might be particularly effective during critical developmental periods. Moreover, a more nuanced analysis of role reversal could investigate its mechanisms, various forms (such as instrumental and emotional parentification), associations, and outcomes in the context of parental mental illness.

Studies that exclusively addressed the coping of children of parents with an addiction disorder were excluded, as research has shown differential effects of parental addiction disorders versus parental mental disorders on the health status of children (Williams & Corrigan, 1992). Nonetheless, investigating coping strategies and their associations with the mental health of offspring of parents with an addiction disorder could be highly interesting.

Finally, exploring how cultural and social norms shape the coping behaviors of offspring of parents with a mental illness could be highly informative. This line of inquiry might help to develop culturally sensitive interventions which are more finely tuned to the needs of children from diverse social and cultural backgrounds. Likewise, although some research has focused on single-parent households (e.g., Brawer-Sherb et al., 2022), expanding studies to include other family structures, such as blended families and children from LGBTQ + families, would provide valuable insights. Are the coping strategies employed by these children similar, or do unique dynamics emerge? Such findings could pave the way for tailored interventions aimed at supporting children in diverse family systems. 

### Concluding Comments

This systematic review is the first to synthesize qualitative findings regarding the coping strategies of children, adolescents, and young adult children of parents with a mental illness. The results revealed that these individuals frequently used support seeking and accommodation as coping strategies, followed by escape and isolation. The system of twelve families of coping by Skinner and Wellborn (1994) provided a useful basis for categorizing the coping instances identified in the included studies. However, it did not fully account for coping behaviors specific to the context of parental mental illness, leading to the creation of a new coping family termed role reversal. Building on these findings, and aiming to address the limitations observed in the hierarchical conceptualization of coping by Skinner and colleagues (2003), a new model of coping for COPMI was proposed. This model not only captures the unique challenges posed by parental mental illness but also provides a comprehensive framework that can be applied to other familial stressors. By integrating insights from related models and encouraging further empirical investigation, the proposed model has the potential to enhance understanding and support for children navigating these complex dynamics. Its adaptability may contribute to the development of targeted interventions, tailored to the specific needs and experiences of children affected by a range of challenging familial circumstances. 

## Supplementary Information

Below is the link to the electronic supplementary material.

Supplementary file1 (DOCX 22 KB)

## Data Availability

The data that support the findings of this systematic review are available from the corresponding author upon reasonable request. This study is a re-analysis of existing data that are publicly available. This study brought together existing research data obtained upon request and subject to license restrictions from a number of different sources.
