# Supplementary material for: Systematic Review and Meta-Synthesis: Coping Strategies of Children, Adolescents, and Young Adults of Parents with a Mental Illness
Source: Clin Child Fam Psychol Rev. 2025 Jul 26;28(3):768–87. doi: 10.1007/s10567-025-00540-8 (PMC12634705; doi:10.1007/s10567-025-00540-8)
Supplement: Supplementary file 1 — Supplementary file1 (DOCX 22 KB) [file 10567_2025_540_MOESM1_ESM.docx]

**Table S 1** Search strategy in Web of Science (All Databases)

| parental mental health | | |  | coping |  | child mental health | | qualitative |
| --- | --- | --- | --- | --- | --- | --- | --- | --- |
|  |  |  | AND |  |  |  | AND |  |
| parent*  maternal  mother*  paternal  father*  caregiver | NEAR/3 | mental illness  mental* ill*  mental disorder*  affective disorder*  mood disorder*  depress*  depressive disorder*  anxiety  anxiety disorder*  stress  distress |  | Coping strategies Coping skills  Coping  Cope  Coping mechanisms Coping behavior | NEAR/3 | emerging adult  adolescent*  teen*  youth  child*  famil* |  | qualitative research  qualitative study  qualitative methods  qualita*  interview  focus group  narrative |

Within a column, search terms were connected with OR

**Table S 2** Study details of included studies

| **No.** | **Author, year,**  **country of origin** | **General study objective** | **Data collection (Data analysis) method** | **Study population** | | | **Parental mental illness** | **Families of coping** |
| --- | --- | --- | --- | --- | --- | --- | --- | --- |
|  |  |  |  | **Sample size (n)** | **% female** | **Age range** |  |  |
| *Qualitative studies* | |  |  |  |  |  |  |  |
| 1. | Brawer-Sherb et al. 2020,  USA | Exploring the lived experiences (impact on various life domains) of emerging adult women who perceive their single mother to be depressed | Interviews | 15 | 100 | 18-25 | Maternal depression | - Self-reliance - Support seeking - Isolation - Helplessness |
| 2. | Garley et al.  1997,  Canada | Exploring the subjective needs, cognitions, and perceptions of asymptomatic children of parents with a mood disorder, to derive relevant content material that would guide the development of a group intervention for this population | Semi-structured focus groups | 6 | 50 | 11-15 | Maternal depression (2), paternal depression (1), paternal manic depression (3) | - Support seeking - Information seeking - Accommodation - Isolation |
| 3. | Kahl & Jungbauer 2014, Germany | Investigating the challenges, resources, coping strategies and needs of children when they have parents affected by schizophrenia | Problem-centered in-depth interviews | 34 | 56 | 8-18 | Schizophrenia (F20 in ICD-10) or schizoaffective disorder (F25 in ICD-10) | - Self-reliance - Support seeking - Problem solving - Accommodation - Isolation - Helplessness - Escape - Submission - Opposition |
| 4. | Küçük Öztürk 2021, Turkey | Explaining the feelings, thoughts, and experiences of a psychiatric nurse whose parents both have a mental illness | Lived experience narrative | 1 | 100 | 30 | Maternal bipolar disorder; paternal schizophrenia | - Self-reliance - Support seeking - Problem solving - Information seeking - Accommodation - Escape |
| 5. | Meadus & Johnson 2000, Canada | Describing the experiences of adolescent children, from their own perspective, of living with a parent suffering from a mood disorder | Unstructured tape-recorded interviews | 3 | 100 | 17 | Bipolar mood disorder (2), unipolar mood disorder (1) | - Support seeking - Information seeking - Accommodation - Isolation - Opposition |
| 6. | Petrowski & Stein 2016, USA | 1) Replicating and extending existing research on perceived role reversal and felt obligation  2) Exploring the nature of family ties between young adult daughters and their fathers and siblings  3) Examining young adults’ accounts of ways that maternal mental illness has impacted their lives | Individual semi-structured interviews | 10 | 100 | 18-22 | Major depression (5), bipolar disorder (3), schizophrenia (2) | - Support seeking - Problem solving - Information seeking - Negotiation - Isolation |
| 7. | Pölkki et al. 2004, Finland | Getting information about the personal experiences of younger and older children of seriously mentally ill parents | Sample 1: text analysis (narratives)  Sample 2: thematic interviews | Sample 1:17  Sample 2: 6 | Sample 1:100  Sample 2: not mentioned | Sample 1: 15-late adulthood; sample 2: 9-11 | Sample 1: examples: psychosis, serious depression  Sample 2: maternal manic depression (5), diagnosis not mentioned (1) | - Support seeking - Accommodation - Submission |
| 8. | Stelling et al. 2008, Germany | Presentation of the life situation of adolescents with mentally ill parents from a developmental psychology perspective and exploration of their stress and need situations | Guideline-based interviews | 15 | 60 | 15-21 | Bipolar disorder (5), unipolar mood disorder (2), schizophrenic spectrum (5), organic psychosis (1), Borderline (1), pseudodementia (Ganser‘s syndrome) (1) | - Self-reliance - Support seeking - Problem solving - Accommodation - Escape |
| 9. | Trondsen 2011, Norway | Providing further insight into the perspectives and experiences of children and adolescents in their present everyday lives with a mentally ill parent | Participant observation of an online self-help group for adolescents with mentally ill parents | 16 | 94 | 15-18 | Severe mental illness, examples: bipolar disorder, psychotic disorder | - Self-reliance - Support seeking - Information seeking - Accommodation - Negotiation - Isolation - Escape |
| 10. | Valdez et al. 2019, USA | Understanding the experiences of Latina/o youth whose mother have depression | Focus groups | 12 | 50 | 9-16 | Maternal depression | - Support seeking - Problem solving - Accommodation |
| 11. | Valiakalayil et al. 2004, Canada | Examining the types of burden described by adolescent children of parents with a diagnosis of schizophrenia | Semi-structured interviews | 13 | 69 | 13-18 | DSM-IV schizophrenia | - Self-reliance - Support seeking - Isolation - Helplessness - Opposition |
| 12. | van Parys et al. 2015, Belgium | Determining how young adults make sense of their childhood experiences of parental depression and exploring how their retrospective reflections could enable us to understand the experiences of children in processes of parentification | Focus-groups | 21 | 86 | 18-29 | Depression (sometimes comorbid with anxiety, alcohol abuse or schizoaffective disorder) | - Self-reliance - Support seeking - Problem solving - Information seeking - Accommodation - Negotiation - Isolation - Escape - Opposition |
| *Mixed methods* | |  |  |  |  |  |  |  |
| 13. | Kuhn & Lenz 2008, Germany | Presentation of the illness-specific stress experience and the coping strategies of children with parents suffering from schizophrenia | Problem-centered guided interviews, survey (SVF-KJ) | 10 | 40 | 8-13 | Schizophrenia (ICD-10: F.2 resp. F.25) | - Support seeking - Problem solving - Isolation - Escape - Opposition |
| 14. | Maybery et al. 2005, Australia | Replicating the findings of Cowling (1999) and Fudge and Mason (2004) and extending current research by examining different viewpoints (child, parent, and professional perspectives) regarding issues facing children whose parents have a mental illness and their needs | Focus groups with children and parents; brief questionnaire for parents, children, mental health professionals | 12 | Not mentioned | 6-16 | Mood disorder (5), anxiety disorder (3), personality disorder (3), psychotic disorder (1) | - Support seeking - Isolation - Escape |
